# Coupled atomistic spin-lattice simulations of ultrafast demagnetization in 3d ferromagnets

**DOI:** 10.1038/s41598-024-58662-y

**Published:** 2024-04-07

**Authors:** M. Pankratova, I. P. Miranda, D. Thonig, M. Pereiro, E. Sjöqvist, A. Delin, P. Scheid, O. Eriksson, A. Bergman

**Affiliations:** 1 Department of Physics and Astronomy, Uppsala University, Box 516, 75120 Uppsala, Sweden; 2 School of Science and Technology, Örebro University, 701 82 Örebro, Sweden; 3 Department of Applied Physics, School of Engineering Sciences, KTH Royal Institute of Technology, AlbaNova University Center, 10691 Stockholm, Sweden; 4 Swedish e-Science Research Center (SeRC), KTH Royal Institute of Technology, 10044 Stockholm, Sweden; 5 Wallenberg Initiative Materials Science for Sustainability (WISE), KTH Royal Institute of Technology, 10044 Stockholm, Sweden; 6 LPCT, CNRS, UMR 7019, BP 70239, Université de Lorraine, 54506 Vandoeuvre-lés-Nancy Cedex, France; 7 IJL, CNRS, UMR 7198, BP 70239, Université de Lorraine, 54000 Nancy Cedex, France; 8 Wallenberg Initiative Materials Science for Sustainability, Uppsala University, 75121 Uppsala, Sweden

**Keywords:** Condensed-matter physics, Theory and computation, Magnetic properties and materials

## Abstract

Despite decades of research, the role of the lattice and its coupling to the magnetisation during ultrafast demagnetisation processes is still not fully understood. Here we report on studies of both explicit and implicit lattice effects on laser induced ultrafast demagnetisation of bcc Fe and fcc Co. We do this using atomistic spin- and lattice dynamics simulations following a heat-conserving three-temperature model. We show that this type of Langevin-based simulation is able to reproduce observed trends of the ultrafast magnetization dynamics of fcc Co and bcc Fe. The parameters used in our models are all obtained from electronic structure theory, with the exception of the lattice dynamics damping term, where a range of parameters were investigated. It was found that while the explicit spin-lattice coupling in the studied systems does not impact the demagnetisation process notably, the lattice damping has a large influence on the details of the magnetization dynamics. The dynamics of Fe and Co following the absorption of a femtosecond laser pulse are compared with previous results for Ni and similarities and differences in the materials’ behavior are analysed. For all elements investigated so far with this model, we obtain a linear relationship between the value of the maximally demagnetized state and the fluence of the laser pulse , which is in agreement with experiments. Moreover, we demonstrate that the demagnetization amplitude is largest for Ni and smallest for Co. This holds over a wide range of the reported electron-phonon couplings, and this demagnetization trend is in agreement with recent experiments.

## Introduction

Ultrafast demagnetization was discovered by Beaurepaire and coauthors in 1996^1^. They observed demagnetization in a nickel film on picosecond timescales following the absorption of a femtosecond laser pulse. From the point of view of applications, ultrafast demagnetization is an important process in all-optical magnetization switching as well as for new applications in magnetic data storage and spintronics^2^. In the same pioneering work of Beuarepaire^1^, these experimental observations were interpreted using a three-temperature model (3TM)^1^, which assumes three thermalized reservoirs, in particular, spin, lattice, and electron reservoirs, that can exchange energy through coupling parameters (electron-phonon, $G_{ep}$, electron-spin, $G_{es}$, and spin-lattice, $G_{sl}$). The 3TM is often used to interpret ultrafast magnetization dynamics processes^1,3,4^. Recently, many other models have been proposed^2,3,5,6^ to describe possible mechanisms of ultrafast demagnetization, such as the importance of spin-dependent transport of laser-excited electrons^7^, the optical intersite spin transfer effect (OISTR) that was considered in Ref.^8^, and the Elliott-Yafet electron-phonon spin-flip scattering, studied in Ref.^9^. Recently, the competition between several ultrafast processes – same-site spin transfer, intersite spin transfer, and ultrafast spin flips—was studied in some detail^10^. In^11^ a dynamic spin-lattice-electron model was proposed. Using this model, the authors calculated laser-induced demagnetization of iron thin film and obtained very accurate agreement with the experimental observations. One of the important outcomes of Ref.^11^ is the establishment of the relations between the dissipative parameters entering the Langevin equations for the lattice and spin degrees of freedom and the subsystems coupling coefficients of 3TM.

In line with those developments, recently Zahn et al.^12,13^ proposed an energy-conserving model based on atomistic spin dynamics simulations. Using this model they studied ultrafast demagnetization of nickel^12^, iron and cobalt^13^, showing a cohesive microscopic picture of the laser-induced dynamics in those 3*d* ferromagnets. Alternatively, our previous work^4^ proposed a heat-conserving three temperature model (HC3TM), where the heat distribution between spin and lattice subsystems is *measured* during the coupled atomistic spin dynamics simulations, in contrast with calculation of temperature in advance. Based on the HC3TM model, Ref.^4^ demonstrated that the fast interplay between spins and electrons allows for a shorter demagnetization time during the laser pulse, when compared to the regular 3TM with constant coefficients, i.e., HC3TM appears to be more consistent with experimental observations for fcc Ni compared to 3TM^1,14,15^. In addition, as argued in Ref.^4^, the HC3TM has the advantage to rely to a much smaller degree on model parameters that can be difficult to estimate, such as, for example, $G_{es}$, $G_{ep}$, and $G_{sl}$ of 3TM. In fact several authors^4,12,13^ report on drastically different 3TM coupling parameters for the here studied systems, and when there is a scatter in such data the reliability of the model becomes questionable.

The impact of laser fluence of ultrafast magnetization dynamics was extensively studied both experimentally and theoretically^16–20^. In particular, it was observed in^19^ for nickel and also for other materials in Refs.^21,22^ that the amplitude of the maximally demagnetized state depends linearly on laser fluence. New experimental results constantly bring new insights and adds to the discussion of existing demagnetization models. As a recent example, Scheid et al.^20^ while experimentally studying maximally demagnetized state of iron, nickel, and cobalt, observed that nickel is the easiest element to demagnetize, however, demagnetization amplitudes for iron and cobalt were almost similar in spite of quite different electronic structures, saturation moments and Curie temperatures. Notably, a linear decrease of the magnetization with the laser fluence contradicts Bloch’s law, stating that the magnetization is proportional to $T_m^{3/2}$, where $T_m$ is the magnon temperature. A model to explain the experimental observations was proposed based on the assumption that the linear dependence of demagnetization on fluence is driven by the increase in temperature, the electron-phonon coupling, the electron-magnon scattering, and a reduction of the interatomic exchange.

In the present work, we further study ultrafast demagnetization of 3*d* ferromagnets with a focus on the role of the lattice dynamics, spin-lattice interaction, and the performance of the recently suggested HC3TM with the strong coupling to ab-initio electronic structure theory. We investigate in full detail the parameter space within the HC3TM model, e.g. by analyzing the impact of the lattice damping, and microscopic spin-lattice coupling during the ultrafast demagnetization. In addition, we apply HC3TM to study the experimentally observed (linear) dependence of the demagnetization amplitude on laser fluence of 3*d* ferromagnets (bcc iron, fcc cobalt, and fcc nickel). The choice of comparing these three elements is natural given the fact that they are the only ferromagnetic transition metal elements, with distinctly different magnetic moments, ordering temperature and how they are understood in terms of being strong or weak ferromagnets.

## Results

### Heat-conserving three-temperature model

**Figure 1:**
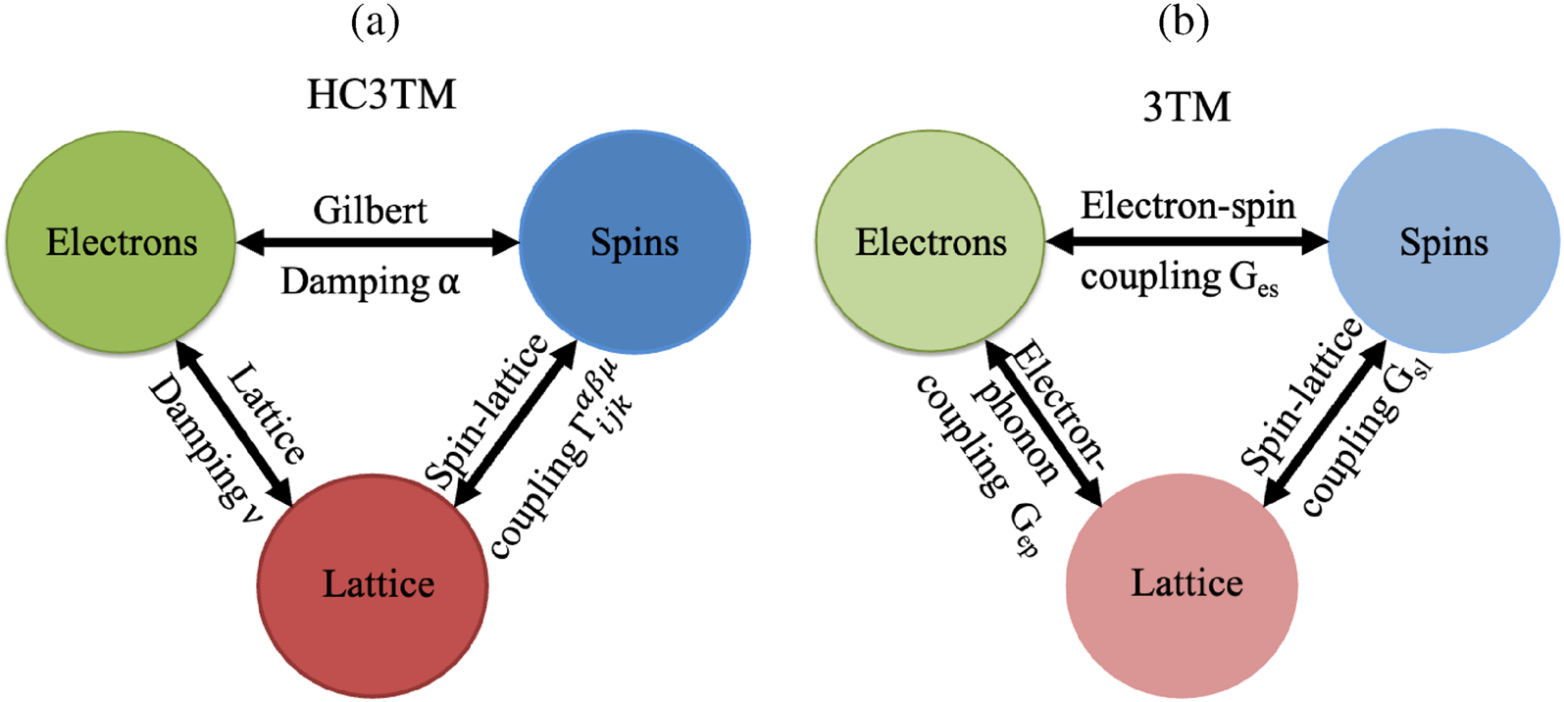
The main idea behind the HC3TM (**a**) and 3TM (**b**) models. The text along the arrows indicates the parameters that drive the heat transfer in each model. For the parameters of the 3TM, see Ref.^1^.

The heat-conserving three-temperature model was proposed in Ref.^4^ for the investigation of ultrafast demagnetization of fcc Ni. Similar to Beurepaire’s 3TM (see Fig. 1a,b), it is assumed that lattice and spin systems are connected to an electronic heat bath. If the electronic reservoir is not considered infinite, the electronic temperature should be decreased as the spin and lattice temperatures increase. This can be modeled by considering the conservation of heat in the system, i.e., the heat that is transferred into the spin and lattice systems is deducted from the electronic system (see Supplementary materials). As mentioned in the Introduction, in the original 3TM formulation the temperature of each subsystem is governed by the coupling coefficients $G_{ep}$, $G_{es}$, and $G_{sl}$^1^.

In the HC3TM formulation, the heat absorbed in the spin and lattice subsystems is simulated on the fly using atomistic spin-lattice dynamics. Thus, the coupling between the subsystems is best described by the atomistic quantities $\nu$ (lattice damping), $\alpha$ (Gilbert damping), and the explicit spin-lattice coupling $\Gamma _{ijk}^{\alpha \beta \mu }$ (see Eq. (8) in Section “Methods”) in the context given by Hellsvik et al.^23^, instead of the microscopic coupling parameters $G_{ep}$, $G_{es}$, $G_{sl}$ of 3TM (see Fig. 1a,b). In that sense, HC3TM allows for a consistent microscopic (and atomistic) description of the model parameters, in a similar fashion as in Ref.^11^.

In the current formulation, all parameters of this theory ($\alpha$^24^, $\nu$^11,25^, and $\Gamma _{ijk}^{\alpha \beta \mu }$^23^) can be calculated using, for example, density functional theory, although in the present work we varied the lattice damping for a range of realistic values, as opposed to performing an explicit calculation of it. The relation between the lattice damping and $G_{ep}$ has been established in^11^ using a spin-lattice-electron three-temperature model. It was shown that the lattice damping is directly proportional to $G_{ep}$, which was previously also reported by Duffy et al. in Refs.^26,27^.

In contrast to 3TM, where the temperatures can be calculated in advance^1^, within the HC3TM, the temperature of the different subsystems is calculated *on-the-fly*—i.e., at every time step of the spin-lattice dynamics simulation—by considering the following relation (see Supplementary materials for motivation):

1 $$\begin{aligned} \Delta T_e (t) = -\frac{C_l(T_l)}{C_e(T_e)}\Delta T_l(t) - \frac{C_s(T_s)}{C_e(T_e)} \Delta T_s(t) + \frac{W(t)}{C_e(T_e)}. \end{aligned}$$

Here, $\Delta$ represents the difference with regard to the temperature, $T_l$ is the lattice temperature, calculated from the average kinetic energy of the lattice vibrations; $\langle E^{kin}_l\rangle /k_B$. The spin temperature, $T_s$, is calculated according to^28^, i.e., using $T_s=\frac{\langle \sum _{i}\left| \hat{\varvec{m}}_{i}\times \varvec{B}_i\right| ^2\rangle }{2k_B\langle \sum _{i}\hat{\varvec{m}}_{i}\cdot \varvec{B}_i\rangle }$, where $\hat{\varvec{m}_{i}}$ is the normalized local spin moment, $\varvec{B}_i$ an effective exchange field (see “Methods” for calculation details). The spin temperature, $T_s$, used in our simulations is defined for an equilibrium situation where the energy flow out of the system is zero. During the ultrafast processes studied in this work, the energy flow is non-zero which raises the question of a reliable definition, and measurement of out-of-equilibrium temperatures^29^. However, in our simulations, we have access to the rate of change of the energy of the spin system, and comparing this rate with the terms that end up defining the spin temperature (Eqs. 15 and 16 in Ref.^28^) we find that it is actually not large enough to notably change the numerical value of the calculated spin temperature. Furthermore, we know from earlier tests that if we translate electron-phonon and electron-magnon couplings from previous 3TM studies to our HC3TM model, we can essentially reproduce the same temperature profiles and demagnetization curves. Together, these two observations support the validity of the use of the equilibrium spin temperature definition (please see Supplementary materials for more information). $C_e(T_e)$, $C_s(T_s)$, and $C_l(T_l)$ in Eq. (1) denote the temperature dependent specific heats of the electronic, spin, and lattice subsystems correspondingly (see Supplementary materials for more information on the heat capacities of the spin, lattice, and electron subsystems and their units). *W*(*t*) represents the impact of a laser pulse, that increases the electronic temperature of Eq. (1), which is proportional to the power source term, modeled as a Gaussian function.

More details concerning the HC3TM and its comparison with 3TM can be found in Ref.^4^ and Supplementary materials.

### HC3TM for cobalt and iron

In this section we present results for spin-lattice dynamics simulations of fcc Co and bcc Fe. In both cases, we run simulations for a $60 \times 60 \times 60$ repetition of the fcc or bcc unit cell with periodic boundary conditions. The simulations are performed for $N_t = 1 \times 10^6$ time steps of $dt = 10^{-16}$s. Other parameters of our simulations are presented in the Table I in Supplementary Materials.

**Figure 2:**
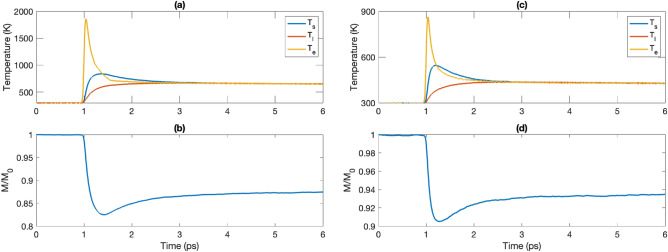
Spin, lattice and electron temperatures of fcc Co (**a)** and bcc Fe (**c**) and magnetization dynamics of fcc Co (**b**), and bcc Fe (**d**) obtained in the HC3TM with fluence of 45 J/m^2^ and 15 J/m^2^ correspondingly.

We start by describing results from ultrafast demagnetization of fcc Co. First, we present temperature profiles (Fig. 2a) for spin, lattice, and electron subsystems, for a laser fluence of 45 J/m^2^, together with the corresponding magnetization dynamics. Other parameters relevant for these simulations are listed in Table I in Supplementary materials. It can be seen from the figure that similar to experimental observations^30^ and theoretical studies^13^ demagnetization happens on subpicosecond timescales, followed by a remagnetization that has a faster recovery after $\sim$ 1 ps and a slower remagnetization after that. We obtain values of the position of the magnetization minima that are close to experimental values, and we also obtain demagnetization/remagnetization timescales that are consistent with experimental observations. It is important to note that in Ref.^13^, using the parametrized three-temperature model, features of the magnetization dynamics similar to the data of Fig. 2b were also observed, however for an adjusted Gilbert damping value of 0.1, which is somewhat unrealistically large^24^. In contrast, in the calculations presented here we use a damping 0.0024, close to experimental value (see Table I in Supplementary materials).

Similar coupled spin-lattice simulations using the HC3TM, were performed for bcc Fe, and shown in Fig. 2c,d. Just like for fcc Co we first present in Fig. 2d magnetization dynamics after the absorption of a laser pulse and the corresponding spin, lattice, and electronic temperatures, see Fig. 2c. Using the HC3TM model we obtain realistic demagnetization/remagnetization times in comparison with experimental studies and in this case even the maximally reduced magnetization is quite close to the experimentally observed value^9^, for the same pulse fluence ($M/M_0\sim 0.9$ in our calculations).

### Results from coupled spin-lattice simulations

To study the impact of direct spin-lattice coupling on the magnetization dynamics (see Eq. (8) in “Methods”) we consider explicitly the spin-lattice coupling, where we obtain the elements of the tensor, $\mathbf {\Gamma }_{ijk}$ in the spin-lattice coupling, from calculations based on density functional theory (e.g., as described in Ref.^23^). Calculated values of these parameters were used to study the impact of direct spin-lattice coupling for bcc Fe, and fcc Co. This explicit, calculated spin-lattice coupling allows for a direct exchange of heat between the spin and lattice subsystems, outside of the channel provided by the HC3TM. Note that the HC3TM can be applied whether or not the direct spin-lattice term (see Eq. (8) in “Methods”) is included or neglected. The results that follow below were obtained from the HC3TM with spin-lattice coupling included explicitly in the simulations and compared with the case without spin-lattice coupling.

**Figure 3:**
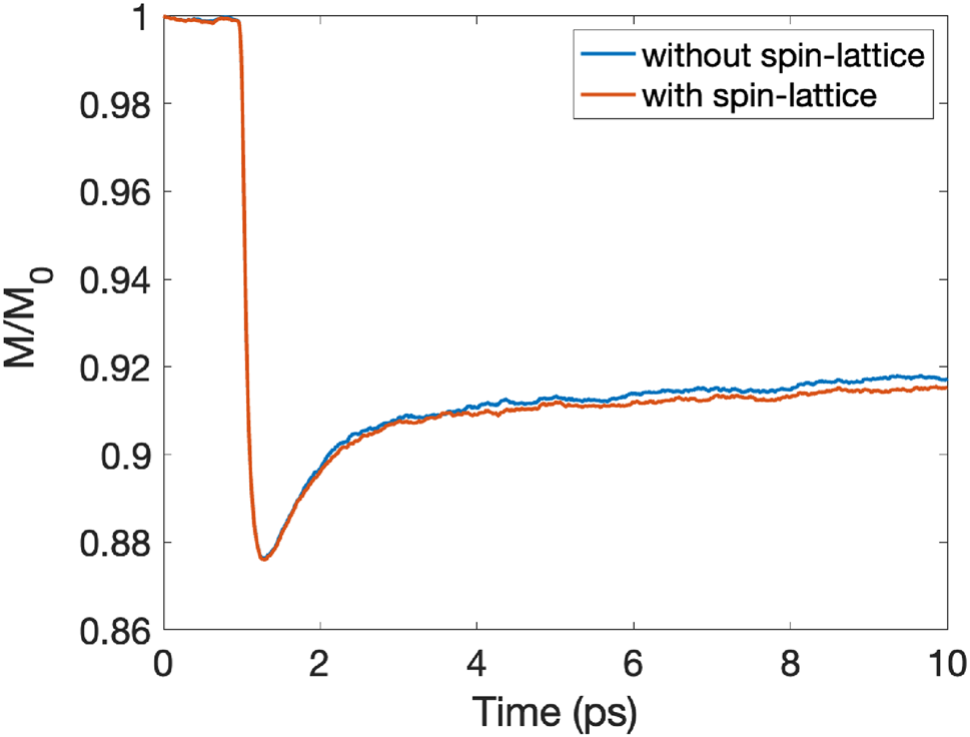
Impact of spin-lattice coupling on magnetization dynamics of bcc Fe. The pulse fluence is 20 J/m^2^.

In Fig. 3 we show the demagnetization profile for bcc Fe from coupled spin-lattice dynamics simulations in combination with the HC3TM. The simulations are, as noted, done with and without the spin-lattice coupling term (given by expression Eq. (8) in “Methods”). It can be seen from Fig. 3 that spin-lattice coupling impacts the magnetization dynamics weakly for bcc Fe. If any effect can be observed, it would be a slightly slower remagnetization rate when the spin-lattice coupling is included. The difference compared to the scenario without the spin-lattice coupling is however almost of the same order of magnitude as the numerical noise of the simulations. When artificially increased by three times, the spin-lattice coupling has a more prominent effect, however, it remains small, as shown in Supplementary materials. We note that a simulation which completely ignores this term still has ability to transfer heat from the spin system to the lattice, via Eq. (1). A possible explanation for the minor influence of the spin-lattice couplings can be sketched as follows: even at the maximum spin temperature in the simulations ($T_s\lesssim 600$ K for Fe), the spin system is far away from the Curie temperature ($T_C$), i.e., with a general strong coupling between spin moments. Thus, the small average displacements (in the order of $\sim 10^{-2}a$) and their distribution throughout the sample result in the summed effect of spin-lattice coupling being only a minor perturbation on the total exchange field. In addition, the spin-lattice couplings considered in this study does not allow the transfer of angular momentum between the spins and the lattice^23^ but since the magnitudes of the calculated spin-lattice couplings are found to be small we do not expect that allowing for a full angular momentum transfer would result a significant deviation from our results.

Also, for bcc Fe the resulting profile is in rather good agreement with experimental observations. For materials with larger spin-lattice coupling (such as CrI_3_^31^) it can be expected that the importance of spin-lattice coupling increases, and our simulations show that for bcc Fe the spin-lattice coupling becomes more important for higher pulse fluences (data not shown).

### Impact of lattice damping

**Figure 4:**
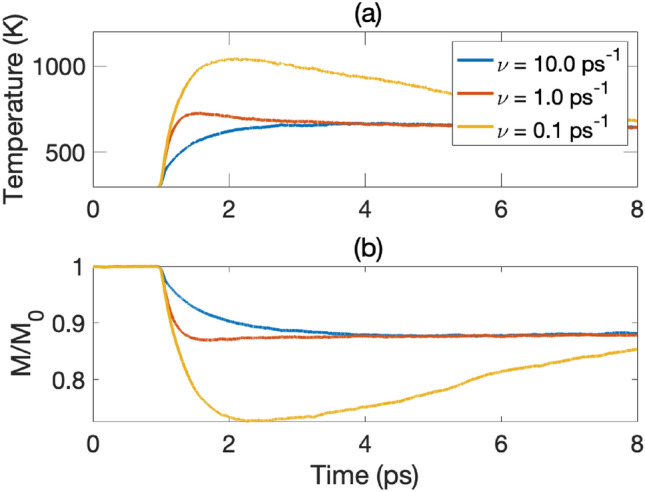
Spin temperature (**a**) and magnetization dynamics (**b**) of fcc Co for various lattice damping values used in HC3TM ($\nu$ in Eq. (4), with temperature-dependent heat capacities (see Ref.^4^ for details). The pulse fluence is 45 J/m^2^.

While the impact of spin-lattice coupling, via Eq. (8), on the magnetization dynamics is only marginal for the elemental 3*d* ferromagnets, the impact of the dynamical properties of the lattice itself, and in particular, the lattice damping, is significant. This is in line with Refs.^12,13,32,33^ (among others), which highlight the importance of the lattice degree of freedom in the ultrafast demagnetization process. We illustrate this for fcc Co, in Fig. 4. Here it is seen that a decrease of the lattice damping ($\nu$ in Eq. (4) leads to an increase of the demagnetization; both the magnetization minimum as well as the reduced magnetization ($M/M_0$) are influenced by the lattice damping parameter. This can be better understood from the fact that the coupled spin and lattice system allows for heat to be dissipated from both reservoirs, when the lattice (and spin) damping parameter is larger. This causes the temperature profile to reach lower values and to equilibrate quicker both for the spin- and lattice system, as shown in Fig. 4, with a resulting stronger impact on the magnetization profile for lower values of the lattice damping. This is detailed clearly when comparing the spin temperatures (Fig. 4) for various values of lattice damping. Lower lattice damping values lead to higher spin temperature and therefore, to larger magnetization drop and a longer remagnetization time (see the dynamics of lattice and electronic temperatures for this case in the Supplementary materials). One of the important outcomes from this observation is that it is essential to take the lattice dynamics properly into account while studying ultrafast demagnetization. If the lattice is not considered at all, then one needs to note, while comparing simulations with experimental data, that this will lead to an overestimation of the magnetization drop and to longer remagnetization times, for a given laser pulse.

Both lattice and Gilbert damping parameters can change as a function of the electronic temperature, and this variation can in principle be captured by *ab-initio* calculations (or, at least, theoretically by using ground-state *ab-initio* quantities), as fair approximations. Let us roughly analyze the magnitudes of these changes. Refs.^12,13^ show that $G_{ep}$ increases $\sim 20\%$ (Ni), $\sim 30\%$ (Co), and $\sim 40\%$ (Fe) between 300 K $\le T_e\lesssim T_{e}^{\text {max}}$ (and so do $\nu$). In turn, $\alpha (T_e)$ changes $\sim 400\%$ in the same temperature interval for Co^34^ (we are assuming that analogous change also happens to Co fcc). The increase of lattice damping will make the heat flow from the electronic system to the lattice system more intensively, so that less heat is redirected to the spin system. This means that we can expect an effect much smaller than the one shown in Fig. 4 coming from $\nu$, and a more intense effect coming from $\alpha (T_e)$ in Co, in the direction of increasing the demagnetization rate (but counterbalanced by $\nu$, as discussed). Therefore, in the investigated systems and fluences, our simple analysis suggests that assuming $\nu$ and $\alpha$ constants is a decent approximation for iron and nickel, however with some changes expected for cobalt.

### Comparison of magnetization dynamics: bcc Fe, fcc Co, fcc Ni

**Figure 5:**
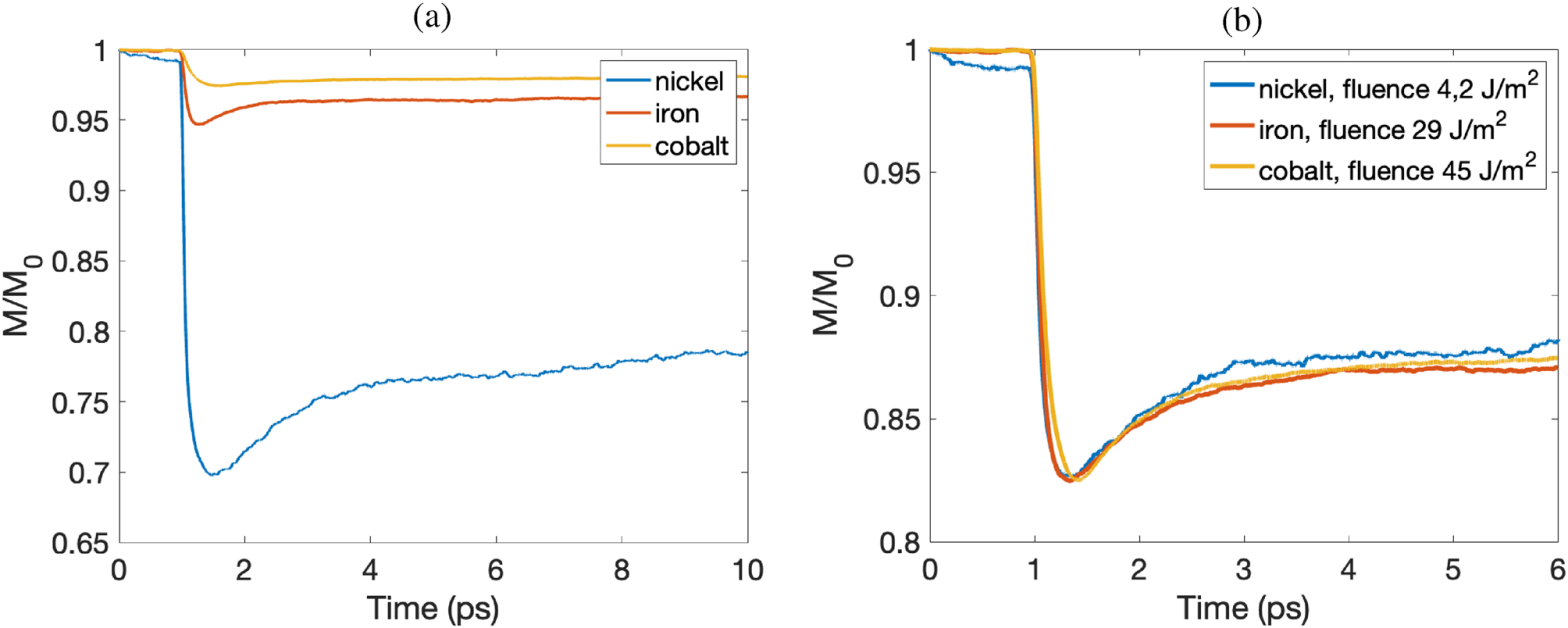
(**a**) Ultrafast demagnetization in fcc Ni, fcc Co and bcc Fe for the same value of the laser pulse fluence 8 J/m^2^. (**b**) Ultrafast demagnetization in fcc Ni, fcc Co and bcc Fe for a different values of laser pulse fluence. The fluence is chosen to obtain the same demagnetization amplitude.

In this section we discuss magnetization dynamics of bcc Fe and fcc Co. We compare the results from the two elements to previous results reported for fcc Ni, which also has been studied using the HC3TM^4^. The results are summarized in Fig. 5. We begin with comparing ultrafast magnetization dynamics of fcc Co, fcc Ni, and bcc Fe, for the same laser pulse fluence, presented in Fig. 5a. It can be seen that in this case, the demagnetization for fcc Ni is the most prominent among the three systems, while the smallest effect is found for fcc Co. This difference in demagnetization properties is consistent with the difference in observed values of $T_C$, which is highest for fcc Co and smallest for fcc Ni.

The next step in our comparison is to choose values of the pulse fluence for all three materials resulting in the same (or very similar) magnetization dynamics. As one can see in Fig. 5b, due to very different $T_C$ values, we need a pulse fluence as high as 45 J/m^2^ for cobalt to exhibit a demagnetization that is similar to nickel for a fluence of 4.2 J/m^2^. It can also be seen from Fig. 5b that in this case the magnetization dynamics of all three systems are very similar. The remaining differences are minute, for example, the minima of cobalt’s magnetization curve appears somewhat later than the one of iron and nickel.

It is important to take into account that this very similar dynamics is observed for specific parameter values, such as the Gilbert and lattice damping. For example, to obtain the curve for cobalt presented in Fig. 5b, we used the damping value from Ref.^35^, see Table I in Supplementary materials. However, with the damping value obtained from *ab-initio* calculations, the magnetization dynamics will be somewhat different. In particular, we then observe much slower remagnetization of cobalt (as, for example, shown in Fig. 4 obtained with the Gilbert damping set to 0.0014). Overall, our findings are in line with studies based on the Landau-Lishits-Bloch approach^19^, where the importance of the coupling between spin and electron systems was concluded.

**Figure 6:**
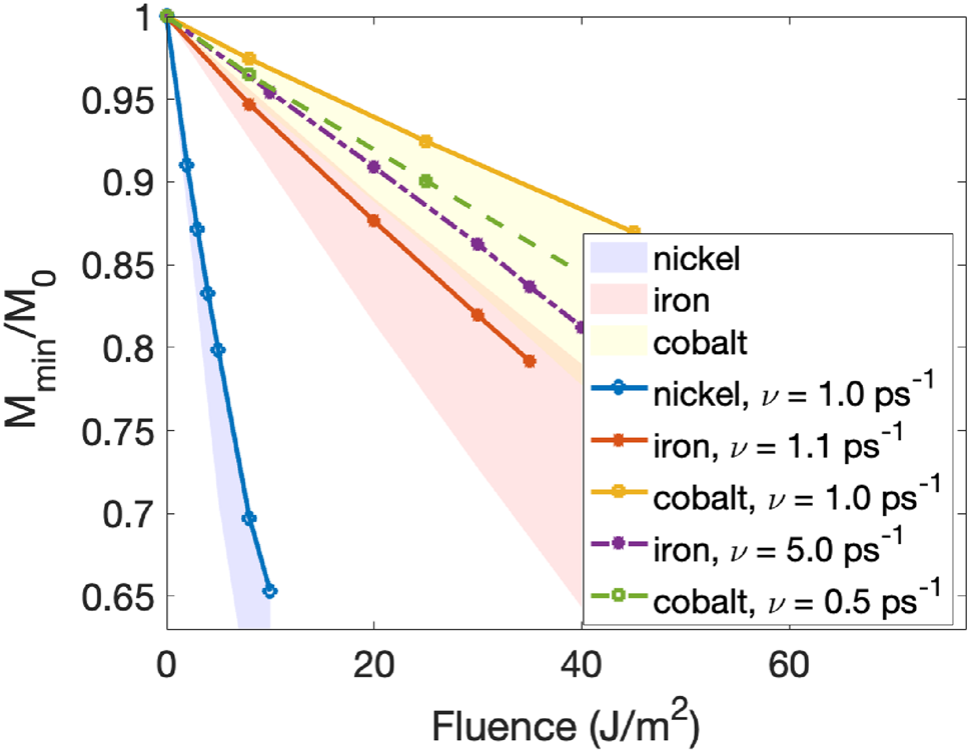
The dependence of the maximally demagnetized value versus laser fluence for bcc Fe, fcc Co and fcc Ni. The shaded area demonstrate a range of reported^4,12,13^ lattice damping parameters obtained using the relation between lattice damping and $G_{ep}$ from^11^.

In Fig. 6 we plot the maximally demagnetized state (minimum value of the magnetization curve, $M_{min}/M_0$) as function of absorbed laser pulse fluence for bcc Fe, fcc Co, and fcc Ni for the same value of the lattice damping (solid lines). The impact of fluence on the demagnetization was reported before for several materials.^16–21^ Here we systematically compare the impact of fluence on 3*d* ferromagnets and study the performance of the HC3TM and the impact of system parameters on the relation between demagnetization and laser fluence. The first observation is that one needs the lowest laser fluence to demagnetize fcc Ni and the strongest for fcc Co. This can, as mentioned earlier in this paper, be connected to the corresponding $T_C$. Secondly, $M_{min}/M_0$ in the simulations depends linearly on laser fluence, something which was observed experimentally in Ref.^20^ and also for other materials in Refs.^21,22^.

In our work we use Gilbert damping that is the highest for nickel and the lowest for cobalt, which might add to the observed trend in Fig. 6. In particular, a higher Gilbert damping, which in the HC3TM can be correlated to the electron-magnon coupling, leads to a stronger heat transfer from the electronic to the spin subsystem, and therefore faster demagnetization, as in the case of nickel.

In the literature, the reported Gilbert damping parameters of iron, nickel, and cobalt deviate between different experiments. For example, reported $\alpha$ values for iron are in the range of 0.0019–0.0072^24^. Nickel does have a consistently larger Gilbert damping than the other two elements, stemming from the $1/m_{Ni}$ scaling and the peak in the minority spin channel at the Fermi level^24^. Varying the Gilbert damping in our HC3TM model results in curves that deviate slightly from those presented in Fig. 6 (data not shown) but the overall behavior still remains the same.

To demonstrate the impact of lattice damping on the trend of the demagnetization more in detail, we have performed spin-lattice simulations for all three elements using several different values of the lattice damping. In addition to the simulations using almost the same lattice damping value ($\nu \approx 1$ ps^-1^—solid lines in Fig. 6), we also used lattice damping values corresponding to the upper and lower limits of the electron-phonon coupling $G_{ep}$ found in the literature^4,12,13^.

These intervals for the reported electron-phonon couplings are shown as the shaded areas in Fig. 6 where it can be noticed that the trend of largest demagnetization for nickel and smallest demagnetization for cobalt holds over almost the full interval of the shaded areas. For all three elements, the lower bounds of the shaded areas in Fig. 6 correspond to the lowest lattice damping while the upper bounds indicate the result of the largest lattice damping of the considered values. This further exemplifies the general picture that a stronger electron-lattice coupling transfers more heat from the electron to the lattice sub-system thus effectively reducing the amount of heat that gets pumped into the electron system.

In Ref.^20^ Scheid et al. experimentally showed that the maximum demagnetization amplitude in Fe and Co are surprisingly similar. Various reasons behind the distribution of demagnetization rates in iron, cobalt, nickel were discussed, including the impact of electron-phonon coupling, the electron-magnon scattering rate, and the ultrafast light-induced quenching of the interatomic exchange. In our model, as seen Fig. 6. this similarity could be explained by a very large lattice damping in Fe relatively to Co. Indeed, in HC3TM, and by using first principle values of Gilbert damping, to obtain similar slopes for iron and cobalt, we need to use a lattice damping for iron 10 times larger than for cobalt. This is illustrated in Fig. 6 where the dashed curves indicate a lattice damping of $\nu \approx 5.0$ ps^-1^ for iron and $\nu \approx 0.5$ ps^-1^ for cobalt. However recent experimental^13^ and first principles^25^ results suggest that the lattice damping in cobalt is in fact larger than in iron. This would amplify the demagnetization in iron, while minimizing the one of cobalt. That conclusion of Scheid et al.^20^ is therefore in line with our findings, because even for the same lattice damping for all systems (which in our model corresponds to electron-phonon coupling, see Fig. 1) we clearly obtain the highest demagnetization rate for nickel followed by iron and then cobalt (see solid lines in Fig. 6). One may further improve the agreement with the experimental observations by considering additionally ultrafast light-induced quenching of the interatomic exchange as suggested in Ref.^20^.

## Discussion and conclusion

Using a heat-conserving three-temperature model we have calculated spin, lattice, and electron temperatures in simulations of the ultrafast magnetization dynamics of fcc cobalt and bcc iron. We have studied in detail the impact of lattice and Gilbert damping and compared results of the magnetization dynamics for bcc Fe and fcc Co with new data for fcc Ni, a system that was previously reported in Ref.^4^. It was found that in studies of ultrafast magnetic phenomena, the lattice dynamics plays a surprisingly important role in the HC3TM, even in cases when the direct spin-lattice coupling is not significant (i.e., when Eq. (8) is neglected). As analyzed here this is caused by how heat is transferred between the different reservoirs in the simulations, and how this heat transfer is influenced by dissipation mechanisms. To be concrete, we find here that the spin temperature used in the simulations depend on several mechanisms, one being how energy is dissipated from the lattice system. An experimental detection of this could possibly be to compare the magnetization dynamics of different isotopes of the systems analyzed here.

Simulations for various laser fluences were performed to study and compare ultrafast demagnetization of iron, cobalt, and nickel in various regimes. We have demonstrated that the simulations are consistent with observations, which show a linear trend between the maximally demagnetized state and laser fluence. We furthermore show that the experimentally found trend that for the same fluence, fcc Ni demagnetizes the most and fcc Co the least, holds for a wide range of realistic choices of the electron-phonon coupling strength.

## Methods. Atomistic spin-lattice dynamics simulations

As reported in Ref.^23^, coupled spin and lattice dynamics can be obtained by solving the following Langevin equations of motion

2 $$\begin{aligned} \frac{d \varvec{m}_i}{d t}= & {} - \frac{\gamma }{(1+\alpha ^2)}\varvec{m}_i\times (\varvec{B}_i+\varvec{B}_i^{fl}) - \frac{\gamma }{(1+\alpha ^2)}\frac{\alpha }{m_i}\varvec{m}_i\times (\varvec{m}_i \times [\varvec{B}_i+\varvec{B}_i^{fl}]) \end{aligned}$$

3 $$\begin{aligned} \frac{d \varvec{u}_k}{dt}= & {} \varvec{v}_k \end{aligned}$$

4 $$\begin{aligned} \frac{d \varvec{v}_k}{dt}= & {} \frac{\varvec{F}_k}{M_k}+\frac{\varvec{F}_k^{fl}}{M_k} - \nu \varvec{v}_k, \end{aligned}$$

where $\varvec{m_i}$ represents an atomic magnetic moment, $m_i$ and $\gamma$ are the saturation magnetization and the gyromagnetic ratio correspondingly. Atomic displacements are denoted by $\varvec{u_k}$, and velocities are $\varvec{v_k}$. We obtain an effective exchange field $\varvec{B}_i = - \partial H_{\textrm{SLD}}/\partial \varvec{m}_i$ from the spin-lattice Hamiltonian, $H_{SLD}$. The Hamiltonian used in this work includes magnetic, lattice and spin-lattice coupling parts following Ref.^23^:

5 $$\begin{aligned} H_{\textrm{SLD}}= H_{\textrm{LL}}+H_{\textrm{SS}}+H_{\textrm{SSL}}. \end{aligned}$$

Here the lattice Hamiltonian reads

6 $$\begin{aligned} H_{\textrm{LL}} = \frac{1}{2} \sum _{kl} \Phi _{kl}^{\mu \nu } u_k^{\mu } u_l^{\nu } + \frac{1}{2} \sum _{k} M_{k} \nu _k^{\mu } \nu _k^{\mu }, \end{aligned}$$

where $\Phi _{kl}^{\mu \nu }$ is the force constant tensor, and $M_k$ is the mass of atom *k*. The magnetic Hamiltonian is described by

7 $$\begin{aligned} H_{\textrm{SS}} = - \frac{1}{2} \sum _{ij} J_{ij}^{\alpha \beta }(0) m_i^{\alpha } m_j^{\beta }, \end{aligned}$$

where $J_{ij}^{\alpha \beta }(0)$ is the exchange tensor at the equilibrium lattice positions. $\alpha ,\beta$ denote Cartesian components in spin space, while $\mu ,\nu$ corresponds to Cartesian components in real space. The magnetic anisotropy is not taken into account in our simulations since for the considered systems it is known to be on $\mu$eV/atom range, and verified to have only a negligible impact on the magnetization dynamics. We note, however, that for materials with larger spin-orbit coupling, the inclusion of an anisotropy term in Eq. (7) may be relevant.

It was shown in^23^ that one of the ways to include spin-lattice coupling is to account for the dependence of exchange interaction values on atomic displacements $\varvec{u_k}$, resulting in functions of the type $J_{ij}^{\alpha \beta }(\varvec{u_k})$ (see Ref.^23^). As the displacements $\varvec{u_k}$ are usually small (see Supplementary materials), one can write the Taylor expansion of the bi-linear magnetic Hamiltonian with respect to the lattice displacements up to the first order, resulting in spin-lattice coupling term bi-linear in spin and linear in displacements^23^. The spin-lattice part of the Hamiltonian can then be written as

8 $$\begin{aligned} H_{\textrm{SSL}} = - \frac{1}{2}\sum _{ijk} \Gamma _{ijk}^{\alpha \beta \mu } m_i^{\alpha } m_j^{\beta } u_k^{\mu }, \end{aligned}$$

where we introduce the coupling constant $\Gamma _{ijk}^{\alpha \beta \mu } =\partial J_{ij}^{\alpha \beta }/\partial u_k^{\mu }$. We note that here only the isotropic part ($\alpha =\beta$) of the resulting tensor $\mathbf {\Gamma }_{ijk}$ is considered for sites $k=i,j$, as the terms for $k\ne i,j$ are at least one order of magnitude smaller^4^. Also note that to allow transfer of angular momentum off-diagonal terms should be included, diagonal terms of tensor $\Gamma _{ijk}^{\alpha \beta \mu }$ lead only to exchange striction^23^. More complete and rotationally invariant Hamiltonian expressions for the atomistic spin-lattice coupling can be found in, e.g.^36,37^.

All parameters for the spin-lattice dynamics simulations were obtained from *ab-initio* calculations (see Supplementary materials) for details). This includes exchange interactions, magnetic moments, inter atomic forces, and spin-lattice couplings. The force at site *k* is defined by $\varvec{F}_k = - \partial H_{\textrm{SLD}}/\partial \varvec{u}_k$. Gilbert and lattice damping constants are denoted $\alpha$ and $\nu$, respectively. In these types of Langevin simulations one employs stochastic fields, $\varvec{B}_i^{fl}$ and $\varvec{F}_{k}^{fl}$, as white noise with properties $\langle B_{i,\mu }^{fl}(t) B_{j,\nu }^{fl}(t') \rangle =2D_M \delta _{ij}\delta _{\mu \nu }\delta (t-t')$ and $\langle F_{i,\mu }^{fl}(t) B_{j,\nu }^{fl}(t') \rangle =2D_L \delta _{kl}\delta _{\mu \nu }\delta (t-t')$. We utilise $D_M= \alpha k_B T_{e}/\gamma m$, $D_L= \nu M k_B T_{e}$, where $k_B$ is Boltzmann constant, and $T_{e}$ is electronic temperature respectively (for details see e.g., Ref.^38^). The formalism above is implemented in the UppASD^39^ code which was used for all simulations in this work.

### Supplementary Information

Supplementary Information.

## Data Availability

The data supporting the findings of this study are available in the paper and upon reasonable request from the corresponding authors (M.Pa. and I.M.).
