# Supplementary material for: Coupled atomistic spin-lattice simulations of ultrafast demagnetization in 3d ferromagnets
Source: Sci Rep. 2024 Apr 7;14:8138. doi: 10.1038/s41598-024-58662-y (PMC10999457; doi:10.1038/s41598-024-58662-y)
Supplement: Supplementary file 1 — Supplementary Information. [file 41598_2024_58662_MOESM1_ESM.pdf]

Supplementary Material
Coupled atomistic spin-lattice simulations of ultrafast demagnetization in $3d$ ferromagnets

M. Pankratova,[1] I. P. Miranda,[1] D. Thonig,[2,1] M. Pereiro,[1] E. Sjöqvist,[1]
A. Delin,[3,4,5] P. Scheid,[6,7] O. Eriksson,[1,2,8] and A. Bergman[1]

[1] *Department of Physics and Astronomy, Uppsala University, Box 516, SE-75120 Uppsala, Sweden*
[2] *School of Science and Technology, Örebro University, SE-701 82, Örebro, Sweden*
[3] *Department of Applied Physics, School of Engineering Sciences, KTH Royal Institute of Technology, AlbaNova University Center, SE-10691 Stockholm, Sweden*
[4] *SeRC (Swedish e-Science Research Center), KTH Royal Institute of Technology, SE-10044 Stockholm, Sweden*
[5] *Wallenberg Initiative Materials Science for Sustainability (WISE), KTH Royal Institute of Technology, SE-10044 Stockholm, Sweden*
[6] *Université de Lorraine, LPCT, CNRS, UMR 7019, BP 70239, 54506 Vandoeuvre-lés-Nancy Cedex, France*
[7] *Université de Lorraine, IJL, CNRS, UMR 7198, BP 70239, 54000 Nancy Cedex, France*
[8] *Wallenberg Initiative Materials Science for Sustainability, Uppsala University, 75121 Uppsala, Sweden*

I. HEAT CAPACITIES OF THE SPIN, LATTICE, AND ELECTRON SUBSYSTEMS

In our calculations we use temperature-dependent heat capacities for the lattice, electron, and spin subsystems. The corresponding curves are presented in Fig. 1.

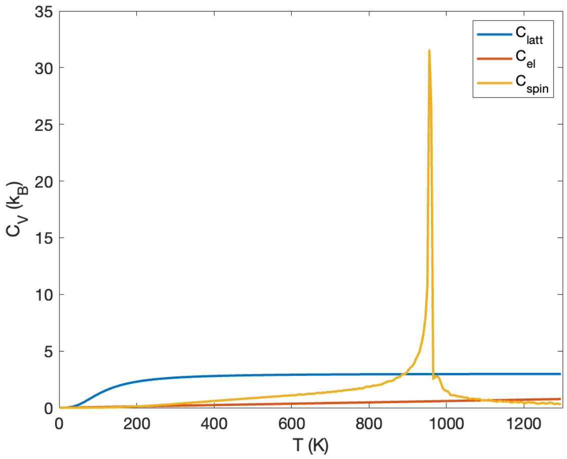

Figure 1. Heat capacities for the electronic, spin, and lattice subsystems used in the calculations for bcc Fe.

The lattice heat capacity is obtained from the Debye model. The spin heat capacity is calculated using a recently proposed approach from quantum statistics [1]. The method more accurately describes heat capacities at low temperatures than what is obtained from Boltzmann statistics. However, its disadvantage is an overestimation of the heat capacity around T_C, as can be seen from Fig. 1. Since in our calculations the spin temperature does not reach T_C, this shortcoming of the method does not impact our results. Moreover, our calculations show that using this approach does not change any of our main conclusions or results. We can confirm that all results and conclusions such as linear dependence of demagnetization amplitude on fluence, etc remain the same regardless of heat capacities used in calculations (please see Appendix A in Ref. [2] for details of heat capacities calculations), including constant (i.e., temperature independent) heat capacities. The only thing that is affected by the change of capacities calculation is demagnetization amplitude as can be seen from Fig.2.

For the electronic subsystem, we assume that the electronic heat capacity is proportional to the electronic temperature $C_e = \gamma_e T_e$, and for bcc Fe we use $\gamma_e = 4.9 \cdot 10^{-3} J\ mol^{-1}\ K^{-2}$.

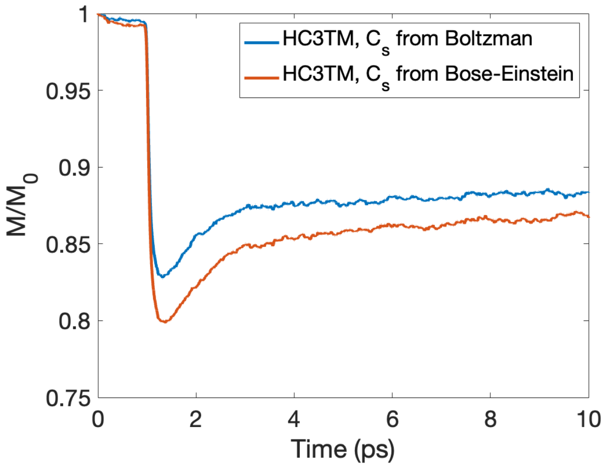

Figure 2. Ultrafast magnetization dynamics of fcc Ni using spin heat capacities calculated either with Boltzmann (blue solid line) or Bose-Einstein statistics (red solid line).

II. DETAILS OF THE *AB-INITIO* CALCULATIONS

Similarly to Ref. [2], the calculation of the interactions used in simulations were performed in the framework of Density Functional Theory (DFT), using two different methods: (i) the real-space linear muffin-tin orbital method in the atomic sphere approximation (RS-LMTO-ASA) [3, 4] and (ii) the plane-wave pseudopotential-based Quantum ESPRESSO (QE) package [5] in combination with the PHONOPY [6] software. On one hand, this choice is motivated, essentially, on the well-known shortcoming of the LMTO-ASA method on obtaining accurate Hellmann-Feynman forces [7] – and, by consequence, phonon frequencies. On the other hand, to calculate the spin-lattice couplings (SLC), a supercell approach is necessary because of the breaking of inversion symmetry and long-range interactions [2]. In this sense, RS-LMTO-ASA is more suitable for this calculation because it deals directly with real-space.

For the computation of the force constants using a combination of QE and PHONOPY, the fcc Ni case used the same definitions as described in Ref. [2]. Analogously, in the case of bcc Fe and fcc Co, in QE the scalar relativistic schemes based on the projector augmented-wave method (PAW) [8] and the ultrasoft pseudopotentials (USPP) [9], respectively, are employed. As the exchange-correlation (XC) functional, we use the generalized gradient approximation (GGA) with the Perdew-Burke-Ernzerhof parametrization [10]. Here, the choice of a spin-polarized GGA XC term is motivated by the fact that the calculated lattice constants as well as the phonon frequencies better reproduce the experimental data in comparison with the local spin density approximation [11]. The iron and cobalt atoms were described by standard pseudopotentials from the QE library, with $3s$, $3p$, $4s$, $4p$ and $3d$ valence electrons. A cutoff of 100 (1080) and 90 (500) Ry for the kinetic energy (charge density) was considered for fcc Co and bcc Fe, in this order. In turn, a Monkhorst-Pack (MP) [12] grid of $24 \times 24 \times 24$ $\vec{k}$-points was set for the first Brillouin zone integration. The self-consistent calculations with a 10^{-10} Ry convergence threshold were carried out using the Marzari-Vanderbilt [13] cold smearing with a spreading of 0.01 Ry. The force constants and phonon frequencies, computed by PHONOPY, were based on $6 \times 6 \times 6$ supercells (216 atoms), where we considered a $4 \times 4 \times 4$ $\vec{k}$-points mesh in the self-consistent cycle.

The exchange parameters, Gilbert damping (α), density of states and spin-lattice couplings (SLC) were obtained using the orthogonal representation [14] of the RS-LMTO-ASA method. In this real-space formalism, the eigenvalue problem is solved with the help of the recursion method [15], where the recursion chain is ended after LL steps by making use of the Beer-Pettifor terminator [16]. For bcc Fe we considered $LL = 31$, while for fcc Co a much higher LL value ($LL = 51$) is needed to better describe the density of states and Green's functions at the Fermi level. In line with our previous work [17], the bulk systems consist of a big cluster in real-space, located in the perfect crystal positions. Differently from the QE calculations (in which the structures were optimized), the lattice parameters were fixed to the experimental values of $a = 2.87$ Å (bcc Fe) and $a = 3.54$ Å (fcc Co) [18]. In this setup, the local density approximation (LSDA), with the von Barth and Hedin parametrization [19], was used. In

particular, this choice of XC was based on the fact that some of quantities that parameterize Eqs. 2-8 were already investigated using LSDA [17, 20–22]. It is a known trait, however, that GGA produces quantities with similar quality for the $3d$ magnetic elements [17, 22, 23]. Finally, the spin-orbit coupling (SOC) is included as a $l \cdot s$ term, computed in each variational step [24, 25].

III. MODEL DERIVATION

We start by assuming the existence of three subsystems as in the three-temperature model (spin s, electrons e, and lattice l), and consider the conservation of energy in the magnetic sample in the absence of any external stimuli:

$$\langle E_e \rangle + \langle E_s \rangle + \langle E_l \rangle = K, \tag{1}$$

where the symbol $\langle\rangle$ represents the ensemble average associated with each subsystem. Here, K is a constant. Therefore, the change of energy in any infinitesimal time dt is given by:

$$\frac{d\langle E_e \rangle}{dt} + \frac{d\langle E_s \rangle}{dt} + \frac{d\langle E_l \rangle}{dt} = 0. \tag{2}$$

For a given temperature (and here we treat temperature as an instantaneous local variable in the context of non-equilibrium thermodynamics, similarly to the treatment in Ref. [26]), the heat capacity of any of the three subsystems can be defined as $C_\lambda = \frac{d\langle E_\lambda \rangle}{dT_\lambda}$ ($\lambda = e, s, l$). Knowing that $C_\lambda \frac{dT_\lambda}{dt} = \frac{d\langle E_\lambda \rangle}{dt}$, from Eq. 2 we have:

$$C_e \frac{dT_e}{dt} + C_s \frac{dT_s}{dt} + C_l \frac{dT_l}{dt} = 0. \tag{3}$$

For a sufficiently small time interval, $\frac{dT_\lambda}{dt} \sim \frac{\Delta T_\lambda}{\Delta t}$, which results in $C_e \Delta T_e + C_s \Delta T_s + C_l \Delta T_l = 0$. However, if a laser pulse is active, injecting an energy density or fluence $E_p(t) = \int_0^t P(t')dt'$ (where $P(t)$ is the pulse power per excited area) into the system, Eq. 1 can be modified to $\langle E_e \rangle + \langle E_s \rangle + \langle E_l \rangle = K + \int_0^t P(t')dt'$. Thus, Eq. 3, under the same small time interval assumption, becomes:

$$\begin{aligned} &C_e \Delta T_e(t) + C_s \Delta T_s(t) + C_l \Delta T_l(t) = P(t)\Delta t \Rightarrow \\ &\Delta T_e(t) = -\frac{C_l}{C_e}\Delta T_l(t) - \frac{C_s}{C_e}\Delta T_s(t) + \frac{P(t)\Delta t}{C_e}, \end{aligned} \tag{4}$$

which is equivalent to Eq.1 of main text with $W(t) \equiv P(t)\Delta t$ (the time dependence of the excess energy) defined. We here follow Refs. [26–29] and the power term $P(t)$ is modeled as a Gaussian of the form $P = P_0 \exp(-(t - t_0)^2/2\sigma^2)$, where t_0 is the center, P_0 is the absorbed pump pulse power amplitude (per area) and σ represents the width of the pulse. Here we consider $\sigma = 0.02$ ps for all simulations. In view of the units of pulse per area, usual in experiments, a fraction of the energy transferred to the (volume) electronic subsystem remains undefined. We here consider a fixed pump penetration depth to convert to volume units, and multiply by the Wigner-Seitz cell volume to match our heat capacity units. We note that our goal here is not to achieve quantitative agreement with experiments, but to explore the parameter space of our model and qualitatively replicate the general linear behavior observed. Thus, parameters like depth can be better explored to align more closely with experimental data on fluences and minimum magnetization.

Using the expression for the electronic temperature above (Eq. 4), we use the following procedure to calculate the spin, electron and lattice temperatures:

1. From Eqs. 2-4 of the main text at every time step

   we calculate lattice and spin temperatures using the expressions $T_l = \langle E_l^{kin} \rangle / k_B$ and $T_s = \frac{\langle \sum_i |\hat{\boldsymbol{m}}_i \times \boldsymbol{B}_i|^2 \rangle}{2k_B \langle \sum_i \hat{\boldsymbol{m}}_i \cdot \boldsymbol{B}_i \rangle}$, where $\hat{\boldsymbol{m}}_i$ is the normalized local spin moment;

2. The increase/decrease of electronic temperature ΔT_e from the initial one (in our case 300 K) is then calculated using Eq. 4 for every time step.

IV. MODEL APPLICABILITY

The approach we use here to calculate spin-lattice coupling is applicable to small atomic displacements.

If we consider the famous Lindemann's melting criterion, which states that the melting of a three-dimensional solid occurs when the root-mean-squared displacement ($\sqrt{|\mathbf{u}_k|^2}(T)$, or RMSD) of each individual particle is $\sim$ 10% of the interparticle distance (from the equilibrium position), then u_k is – by definition – usually small. Bringing this concept to a more realistic and material-centered scenario, one can calculate the $\sqrt{u^2_{k_\gamma}}(T)$ for each Cartesian direction $\gamma = \{x, y, z\}$ via the thermal displacements implementation in PHONOPY [6], which, in turn, uses the theoretical force constants obtained by DFT. Figure 3 shows the obtained phonon dispersion and RMSD for bcc Fe, together with a comparison with experiments.

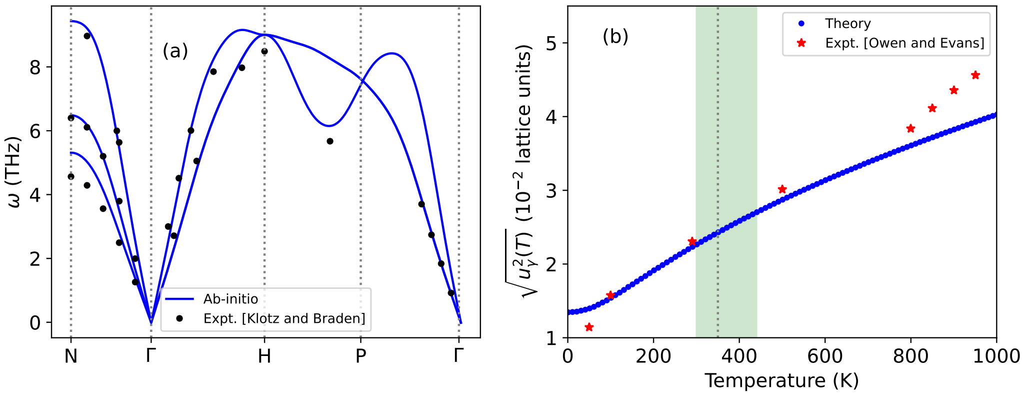

Figure 3. Calculated properties of bcc Fe: (a) phonon dispersion; and (b) root-mean-squared displacement (RMSD) as a function of temperature. In both, theoretical results are compared with experimental data, extracted from Ref. [30] (phonons) and Ref. [31] (mean square displacements). The green region in panel (b) indicates the lattice temperature interval in our simulations. Note that the data from the table in Ref. [31] was divided by $\sqrt{3}$ as we are plotting only one of the Cartesian components.

Despite some anharmonicity observed at high temperatures ($T > 800$ K), it is clear from Fig. 3 that the Cartesian-decomposed RMSD's are definitely lower than $0.05a$ in the temperature range shown (which gives an upper bound of $\sim 0.08a$ for the full RMSD) – where a is the bulk lattice parameter. In other words, u_k is small enough for the spin-lattice Hamiltonian to be a reasonable approximation at all simulated temperatures. The situation is similar for fcc Ni [2] and fcc Co (not shown).

A. Applicability of spin temperatures definition

In the main text, it is suggested that, even in a non-equilibrium situation, the explicit expression derived in Ref. [32] for a dynamic spin ensemble is still robust to monitor the temperature of the spin subsystem at all times. This issue was to some extent discussed in Ref.[33], for similar simulations as what is presented here, but for bcc Co. In Ref.[33] it was found that the distribution of atomic magnetic moments follows a Boltzman distribution function, even in the transient state caused by an intense laser fluence, over the entire simulation interval of 0 - 1 ps (see Fig.10 of Ref. [33]), which supports the assumption of connecting a temperature to a distribution of magnetic moments (as is done in the present work).

Another, straightforward, way to investigate this, is to check how the simulated $\bar{m}_s(t)$ and $T_s^{\text{dynamic}}(t)$ during the ultrafast dynamics compares with data from an equilibrium situation of the spin subsystem. In the latter case, both magnetism and in particular temperature, T_s^{eq}, are easy to define. From curves like the ones shown in Fig.2 of the main part of this communication, it is easy to identify a time dependent magnetic moment, $\bar{m}_s(t)$, as well as a time dependent, dynamical temperature, $T_s^{\text{dynamic}}(t)$. This enables a dynamic relationship between magnetization, $\bar{m}_s(t)$, and temperature, $T_s^{\text{dynamic}}(t)$,

that can be compared to the static M versus T curve. Figure 4 illustrates such a comparison, with a relationship between the simulated points $(\bar{m}_s(t), T_s^{\mathrm{dynamic}}(t))$ (red and yellow/green dots), and the static magnetization curve (blue line) for bcc Fe. Note that the red dot marks the value of $(\bar{m}_s(t), T_s^{\mathrm{dynamic}}(t))$ just before a pulse with a fluence of 20 J/m^2, is applied. The yellow/green dots then follow the curve carved out by $(\bar{m}_s(t), T_s^{\mathrm{dynamic}}(t))$ over a time interval of 1 ps. Note that the color scale of this curve indicates the time of the simulation, with yellow being earlier and green indicating later times. It can be seen from the figure that the curve defined by $(\bar{m}_s(t), T_s^{\mathrm{dynamic}}(t))$ lies surprisingly close to the static M vs T curve, which speaks for the validity of defining a spin-temperature as outlined in the main part of this communication (see in particular the discussion below Eqn.1) and which is also consistent with the analysis of Chimata et al. [33]. It is however important to notice that, although the considered fluence and simulated results lead to a reasonably physically consistent concept of a dynamical temperature, $T_s^{\mathrm{dynamic}}(t)$), this method should be applied with caution for arbitrarily large fluences, where it may result in large deviations from equilibrium temperatures. Naturally, such situation would be interesting to study experimentally as well as from simulations.

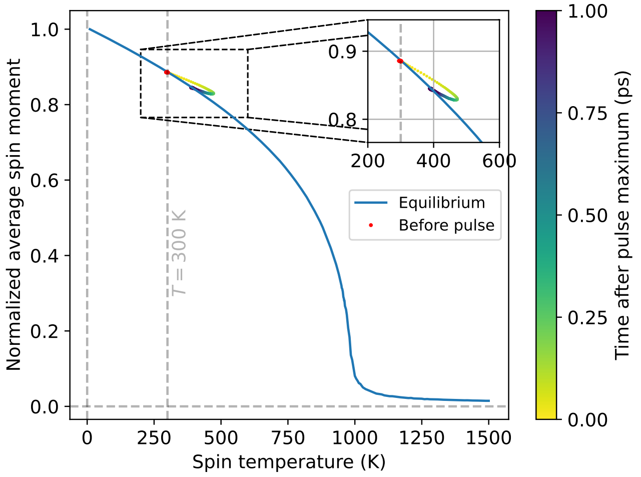

Figure 4. The temperature-dependent magnetization curve of bcc Fe (blue line) and its relation to the simulated $(\bar{m}_s(t), T_s^{\mathrm{out}}(t))$ points (red and yellow to dark blue dots) during the ultrafast dynamics for a fluence of 20 J/m^2. Red dots mark the interval before the pulse, while yellow/ to dark blue dots represent the post-pulse period. The color bar indicates the time after the pulse maximum.

V. PARAMETERS USED IN THE SIMULATIONS

Table I. List of the parameters used in the simulations.

Parameter	Value
Iron	
Gilbert damping α	0.008 [26]
Lattice damping ν	1.1 ps^{-1}
Cobalt	
Gilbert damping α	0.0024 ([34]) or 0.0014 [17]
Lattice damping ν	1.0 ps^{-1}
Nickel	
Gilbert damping α	0.024 ([2])
Lattice damping ν	1.0 ps^{-1}

VI. IMPACT OF THE LATTICE DAMPING AND SPIN-LATTICE COUPLING

In Fig.4 of the main text the magnetization dynamics of fccCo is given for various values of the lattice damping with corresponding dynamics of spin temperature T_s. Here, in Fig.5 we present for the same simulations, in addition, the dynamics of corresponding electronic and lattice temperatures as a ratio T_e/T_l.

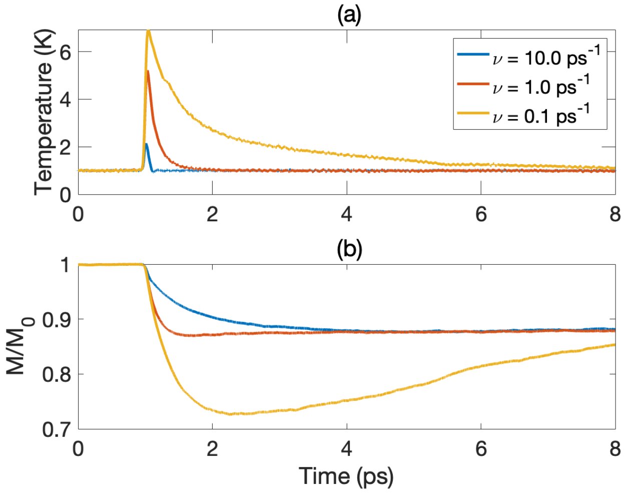

Figure 5. Dynamics of electronic by lattice temperature T_e/T_l (a) and the corresponding ultrafast magnetization dynamics (b) and of fcc Co for various values of lattice damping.

It is demonstrated in the main text that calculated from ab-initio spin-lattice coupling has only marginal impact on magnetization dynamics of bcc Fe. Figure 6 shows the impact of the spin-lattice coupling artificially increased by three times in bcc Fe from the *ab-initio* values. In that case, the impact becomes slightly more evident, but it still can be classified as a perturbative effect.

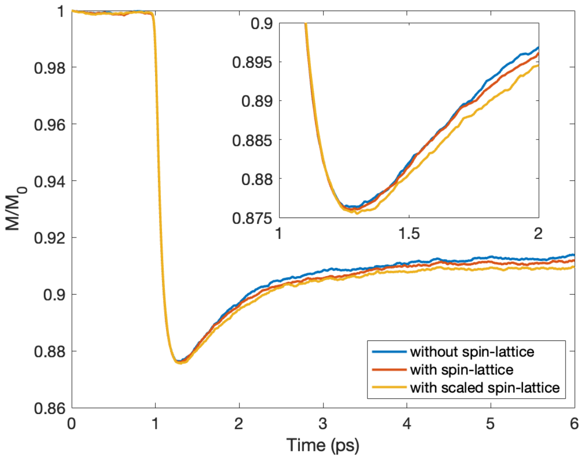

Figure 6. The impact of the *ab-initio* spin-lattice coupling (red curve) in bcc Fe. The calculated coupling values were artificially increased three times to demonstrate the effect (yellow curve). The insert shows the magnified area of the magnetization dynamics near the demagnetization curve minimum.

[1] L. Bergqvist and A. Bergman, Realistic finite temperature simulations of magnetic systems using quantum statistics, Phys. Rev. Materials **2**, 013802 (2018).
[2] M. Pankratova, I. P. Miranda, D. Thonig, M. Pereiro, E. Sjöqvist, A. Delin, O. Eriksson, and A. Bergman, Heat-conserving three-temperature model for ultrafast demagnetization in nickel, Phys. Rev. B **106**, 174407 (2022).
[3] P. R. Peduto, S. Frota-Pessa, and M. S. Methfessel, First-principles linear muffin-tin orbital atomic-sphere approximation calculations in real space, Phys. Rev. B **44**, 13283 (1991).
[4] S. Frota-Pessoa, First-principles real-space linear-muffin-tin-orbital calculations of 3d impurities in cu, Phys. Rev. B **46**, 14570 (1992).
[5] P. Giannozzi, O. Andreussi, T. Brumme, O. Bunau, M. B. Nardelli, M. Calandra, R. Car, C. Cavazzoni, D. Ceresoli, M. Cococcioni, *et al.*, Advanced capabilities for materials modelling with quantum espresso, Journal of physics: Condensed matter **29**, 465901 (2017).
[6] A. Togo and I. Tanaka, First principles phonon calculations in materials science, Scr. Mater. **108**, 1 (2015).
[7] J. M. Wills, M. Alouani, P. Andersson, A. Delin, O. Eriksson, and O. Grechnyev, Linear muffin-tin orbital method in the atomic sphere approximation, in *Full-Potential Electronic Structure Method: Energy and Force Calculations with Density Functional and Dynamical Mean Field Theory* (Springer Berlin Heidelberg, Berlin, Heidelberg, 2010) pp. 35–46.
[8] P. E. Blöchl, Projector augmented-wave method, Phys. Rev. B **50**, 17953 (1994).
[9] D. Vanderbilt, Soft self-consistent pseudopotentials in a generalized eigenvalue formalism, Phys. Rev. B **41**, 7892 (1990).
[10] J. P. Perdew, K. Burke, and M. Ernzerhof, Generalized gradient approximation made simple, Phys. Rev. Lett. **77**, 3865 (1996).
[11] A. Dal Corso and S. de Gironcoli, Ab initio phonon dispersions of fe and ni, Phys. Rev. B **62**, 273 (2000).
[12] H. J. Monkhorst and J. D. Pack, Special points for brillouin-zone integrations, Phys. Rev. B **13**, 5188 (1976).
[13] N. Marzari, D. Vanderbilt, A. De Vita, and M. C. Payne, Thermal contraction and disordering of the al(110) surface, Phys. Rev. Lett. **82**, 3296 (1999).
[14] S. B. Legoas, A. A. Araujo, B. Laks, A. B. Klautau, and S. Frota-Pessôa, Self-consistent electronic structure of mo(001) and w(001) surfaces, Phys. Rev. B **61**, 10417 (2000).
[15] R. Haydock, The recursive solution of the schrodinger equation, in *Solid state physics*, Vol. 35 (Elsevier, 1980) pp. 215–294.
[16] N. Beer and D. Pettifor, The recursion method and the estimation of local densities of states, in *The Electronic Structure of Complex Systems* (Springer, 1984) pp. 769–777.
[17] Z. Lu, I. P. Miranda, S. Streib, M. Pereiro, E. Sjöqvist, O. Eriksson, A. Bergman, D. Thonig, and A. Delin, Influence of nonlocal damping on magnon properties of ferromagnets, Phys. Rev. B **108**, 014433 (2023).
[18] B. H. Billins and D. E. Gray, eds., *American Institute of Physics Handbook (3rd Ed)* (McGraw-Hill, 1972).
[19] U. Von Barth and L. Hedin, A local exchange-correlation potential for the spin polarized case. i, Journal of Physics C: Solid State Physics **5**, 1629 (1972).
[20] M. Pajda, J. Kudrnovský, I. Turek, V. Drchal, and P. Bruno, Ab initio calculations of exchange interactions, spin-wave stiffness constants, and curie temperatures of fe, co, and ni, Phys. Rev. B **64**, 174402 (2001).
[21] Y. O. Kvashnin, O. Grånäs, I. Di Marco, M. I. Katsnelson, A. I. Lichtenstein, and O. Eriksson, Exchange parameters of strongly correlated materials: Extraction from spin-polarized density functional theory plus dynamical mean-field theory, Phys. Rev. B **91**, 125133 (2015).
[22] A. Szilva, Y. Kvashnin, E. A. Stepanov, L. Nordström, O. Eriksson, A. I. Lichtenstein, and M. I. Katsnelson, Quantitative theory of magnetic interactions in solids, Rev. Mod. Phys. **95**, 035004 (2023).
[23] A. Grechnev, I. Di Marco, M. I. Katsnelson, A. I. Lichtenstein, J. Wills, and O. Eriksson, Theory of bulk and surface quasiparticle spectra for fe, co, and ni, Phys. Rev. B **76**, 035107 (2007).
[24] O. K. Andersen, Linear methods in band theory, Phys. Rev. B **12**, 3060 (1975).
[25] S. Frota-Pessôa, Magnetic behavior of 3*d* impurities in cu, ag, and au: First-principles calculations of orbital moments, Phys. Rev. B **69**, 104401 (2004).
[26] P.-W. Ma, S. L. Dudarev, and C. H. Woo, Spin-lattice-electron dynamics simulations of magnetic materials, Phys. Rev. B **85**, 184301 (2012).
[27] D. Zahn, F. Jakobs, Y. W. Windsor, H. Seiler, T. Vasileiadis, T. A. Butcher, Y. Qi, D. Engel, U. Atxitia, J. Vorberger, and R. Ernstorfer, Lattice dynamics and ultrafast energy flow between electrons, spins, and phonons in a 3d ferromagnet, Phys. Rev. Research **3**, 023032 (2021).
[28] D. Zahn, F. Jakobs, H. Seiler, T. A. Butcher, D. Engel, J. Vorberger, U. Atxitia, Y. W. Windsor, and R. Ernstorfer, Intrinsic energy flow in laser-excited 3*d* ferromagnets, Phys. Rev. Research **4**, 013104 (2022).
[29] P. Maldonado, K. Carva, M. Flammer, and P. M. Oppeneer, Theory of out-of-equilibrium ultrafast relaxation dynamics in metals, Phys. Rev. B **96**, 174439 (2017).
[30] S. Klotz and M. Braden, Phonon dispersion of bcc iron to 10 gpa, Phys. Rev. Lett. **85**, 3209 (2000).
[31] E. A. Owen and E. W. Evans, The variation with temperature of atomic vibration amplitudes in iron, Br. J. Appl. Phys. **18**, 611 (1967).

[32] P.-W. Ma, S. L. Dudarev, A. A. Semenov, and C. H. Woo, Temperature for a dynamic spin ensemble, Phys. Rev. E **82**, 031111 (2010).

[33] R. Chimata, E. K. Delczeg-Czirjak, A. Szilva, R. Cardias, Y. O. Kvashnin, M. Pereiro, S. Mankovsky, H. Ebert, D. Thonig, B. Sanyal, A. B. Klautau, and O. Eriksson, Magnetism and ultrafast magnetization dynamics of co and comn alloys at finite temperature, Phys. Rev. B **95**, 214417 (2017).

[34] M. A. W. Schoen, J. Lucassen, H. T. Nembach, B. Koopmans, T. J. Silva, C. H. Back, and J. M. Shaw, Magnetic properties in ultrathin $3d$ transition-metal binary alloys. ii. experimental verification of quantitative theories of damping and spin pumping, Phys. Rev. B **95**, 134411 (2017).
